# The Impact of Public Health Expenditure on Health Outcomes in South Africa

**DOI:** 10.3390/ijerph16162993

**Published:** 2019-08-20

**Authors:** Besuthu Hlafa, Kin Sibanda, Dumisani MacDonald Hompashe

**Affiliations:** Department of Economics, University of Fort Hare, Alice 5700, South Africa

**Keywords:** life expectancy, under-5 mortality, public health expenditure, fixed effect, random effect, seemingly unrelated regression, feasible generalized least of squares, South Africa

## Abstract

Health holds an important position in maintaining economic development since it is both a prerequisite for and an outcome of economic development. This means that health contributes greatly to the attainment of sustainable development and health outcomes. The importance of health is demonstrated in the Millennium Development Goals (MDGs) where three of the eight goals are aimed at improving health outcomes. Despite progress made by other middle-income countries in achieving health-related MDGs, South Africa is still worse off in respect of health outcomes and experiences a challenge in attaining positive outcomes for these goals. This study’s main focus was to identify the association between public health expenditure and health outcomes in South Africa’s nine provinces from 2002 to 2016. The study implemented fixed effects and a random effects panel data estimation technique to control for time effects and individual provincial heterogeneity. This was followed by employing the Hausman specification test to identify the fixed effects model as the appropriate estimator for the study. The study also employed the seemingly unrelated regression (SUR) model and the least squares dummy variable (LSDV) model to examine the impact of public health expenditure on each province separately. The findings elucidated that the relationship between public health expenditure and health outcomes in South Africa varied across provinces depending on provincial management and infrastructure availability.

## 1. Introduction

The democratic South African government in 1994 had one task, among many, to provide primary healthcare (PHC) for every South African. The government’s central task was to address the disempowerment, discrimination, and underdevelopment that had weakened the healthcare system over the past few decades [1]. According to the World Health Organization, access to basic healthcare can be described in terms of financial coverage, which refers to social protection from financial and socio-economic challenges of access to health, population coverage, and service coverage. The restructuring of the public healthcare sector enabled some improvements in public health care, which included improved access to medical resources for everyone, rationalisation of health management and a more equitable health expenditure [2]. The African National Congress (ANC) published a health plan which signified a model for changes that were desired in the post-Apartheid era [3]. The health plan encompassed free health services for children under the age of six years and pregnant mothers. The Redistribution and Development Programme (RDP), which was the government’s primary socio-economic program, recognized the importance and value of PHC as an essential part of its strategy [4]. The new government had thus succeeded in addressing some of the areas of interest in the healthcare system in post-1994 South Africa.

The South African constitution is binding to the state in the realisation of the right to basic health care, and yet the burden of HIV/AIDS resulted in some erosion of the aforementioned post-1994 gains and improvements in public healthcare. Despite the 1994 health reforms, the burden of diseases increased fourfold from non-communicable diseases/chronic diseases to communicable diseases such as HIV/AIDS and sexually transmitted infections (STIs). Additionally, violence and injury led to increased mortality rates, which resulted in the loss of healthy lives [1].

Literature has, to some extent, discussed the relationship between public health expenditure and related health outcomes. Panel data estimation techniques have mostly been used in the literature to investigate this aforementioned relationship. However, this has primarily been implemented at country level. Thus, this paper sought to investigate the impact of public health expenditure on health outcomes at a provincial level in South Africa using panel data analysis. Moreover, this study used robust estimators (SCC and FGLS) to econometrically investigate this impact and to also ensure consistent and efficient inferences. For efficient provincial analysis, this paper used SUR and LSDV.

## 2. Public Health Expenditure and Related Health Outcomes—An Overview

The 2017 South African Health Review (SAHR) has captured several points of view on various programmatic activities that were intended to strengthen the health system [5]. Among these viewpoints are issues encapsulated in the Ministry of Health’s arranged service delivery agreement, which related to increasing life expectancy, decreasing child mortality, combating HIV and AIDS and decreasing the burden of sickness from both chronic and non-chronic diseases. The pledge to PHC re-engineering by the South African Department of Health (DoH) has been driven by a fourfold burden of disease, which has been fueled by a range of hazardous factors, including poor diets, STIs resulting from unsafe sex, alcohol abuse which triggers interpersonal violence and maternal and childhood malnutrition. Tollman and Rispel (1995) (as cited in [5]) also highlighted a number of issues on governance and leadership. Amongst those problems highlighted, one that still affects the South African health system is the miscommunication, or as Tollman and Rispel (1995) called it, the tension between national and provincial departments of health. The lines of communication between these two departmental levels can be catastrophic if left unchecked and, therefore, an improvement in this regard needs to be encouraged. South African public healthcare is financed through tax revenues [6]. Public healthcare is provided to people through public hospitals and clinics in rural areas. Public health systems provide virtually universal coverage, i.e., all citizens are entitled to use the widely distributed service points and primary healthcare services are free at the point of service [7]. The Health Systems Trust (HST) (2014), as cited in [5], highlighted that 82% of the population depends on the provision of public healthcare. The provision of public healthcare falls directly under the provincial spheres of government and within those spheres, 94.4% of the consolidated government health expenditure in the 2014/15 financial year was spent by provinces [6]. The preceding information emphasizes the importance of public healthcare services in South Africa and also the number of people that depend on those services. The level of consolidated government expenditure spent by provinces should suggest an improvement in public healthcare services and yet, that is not the case [6]. With respect to health, the role of local government is limited to environmental health and also to emergency medical health services [8]. On the other hand, the policies formulated by the DoH are implemented at a provincial level, which confirms that to a large extent, provinces are responsible for the healthcare of the country through expenditures. The main funding instruments of South African public healthcare are conditional and unconditional fiscal transfers from the national government to the nine provinces, but public healthcare services are mainly funded by the unconditional grants [6].

### Expenditure Trends and Health Outcomes

Public health expenditure has been increasing provisionally over the past decade, and the reason behind that was solely towards improving health access to previously disadvantaged South Africans [5]. There is an inverse relationship between public health expenditure and the under-five mortality rate in South Africa, as the increase in public health expenditure is not accompanied by a lowering of the under-five mortality rate [6]. Moreover, [8](as cited in [6] stated that one of the good indicators of availability, utilization, and effectiveness of healthcare is under-five mortality rate. In the case of South Africa, it could be possible that there is a lack of efficiency in terms of resource usage, leading to the condition of an inverse relationship between the under-five mortality rate and public health expenditure in South Africa [6].

By the end of the 2014 financial year, the total public health expenditure was measured at 9% of gross domestic product and has since been continuously increasing [9]. It was also highlighted that corruption has proven to be a problem in the South African public health sector [9]. This is reflected by the worsening health outcomes failing to reflect the allocated budget. However, it is unfortunate that there are no globally validated indicators to measure this corruption, for it would have been easy to find ways of eradication [9].

**Figure 1:**
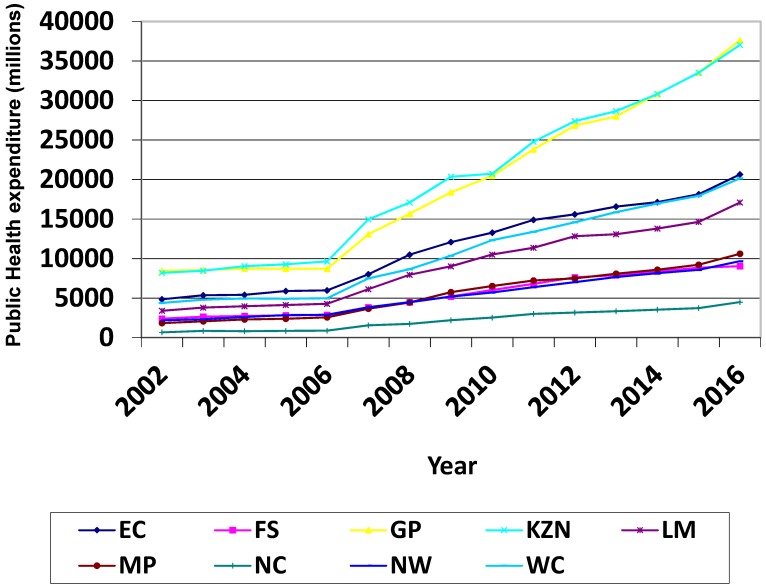
Provincial health expenditure. Source: Own Graph Made with Data from SAHR Reports and STATSA Reports.

The effects of the 2008 recession were delayed in South Africa until around 2011/12 where a sluggish growth in expenditure is displayed in Figure 1 [5]. The authors attributed this to government-adopted counter-cyclical fiscal policy of which the point is to secure social spending and stimulate economic growth. Though the ramifications of the 2008 recession were only later displayed in the South African health sector, government spending has continued to increase substantially up to date [5].

One of the reasons why increased public health expenditure is not reflected by health outcomes is due to control of personnel costs by the government. As elucidated by [5], personnel numbers have been limited to control personnel expenditure and this is due to the apparent powerlessness of government to control personnel unit costs. In most South African provinces, some forms of restrictions in filling up vacant posts for medical personnel have been imposed with the intention of cutting personnel costs [5]. Having a decreasing ratio of physicians (RoP) per 100,000 population is undesirable for South Africa as this threatens the hope of achieving improved health outcomes.

It has also been reported [5] that the number of employees in the provincial departments of health increased by 80,679 from 2012 to 2016. However, this does not necessarily say in which directorates within the provincial departments the employees belong, leading to uncertainties as to whether the number of medical practitioners increased as well, and if it did, to what extent. Payment of employees comprises an increasingly large proportion of provincial health budgets. Health personnel costs have expanded from 57.2% of total expenditure in 2008/09 to 63.4% in 2016/17.

The 2016/17 annual report of the KwaZulu-Natal (KZN) department of health highlighted that despite the challenges that the province faced pertaining to healthcare, considerable levels of success in reducing the burden of disease were registered [10]. Significant reductions in maternal, infant, and child mortalities were noted in line with the National Development Plan (NDP) goals [10]. Higher expenditures depicted in Figure 1 for the KZN and Gauteng province represent efforts of combating the burden of disease to provide better health outcomes in these provinces. Literature [8,10,11] highlighted improvements in some health outcomes because of these high expenditures.

The Gauteng provincial 2016/17 annual report noted a mixed bag of challenges and successes in their health department. The increased expenditure for the Gauteng province as depicted in Figure 1 led to positive health outcomes [11]. As a result of such expenditures, the life expectancy rate as depicted in the Gauteng annual report for the year 2016 was 63.3 years, being above the national average of 62.4 years.

## 3. A Theoretical Framework of Health Expenditure and Health Outcomes

The theoretical framework utilized in the paper is the principal-agent problem and the Grossman’s (1972) model of health as derived demand. The Grossman (1972) model is derived from the model of optimal control, where health capital is a consumption good as well as an investment good and it has been argued to be amongst the major contributors to the theoretical framework of health economics [12]. This model made an important contribution to the literature by separating the biological health production function from the behavioral input demand function, allowing for the use of socio-economic inputs such as income and government expenditure in the production function estimation [6]. This implies that socio-economic inputs such as public health expenditure do not have a direct impact on health outcomes. However, these inputs indirectly impact health outcomes through proximate determinants, such that health expenditures provide resources to purchase new medicine, leading to better outcomes. Moreover, literacy as an input helps improve health outcomes in the sense that through literacy, proper usage and dosage of medicine may also help improve the quality of life. This forms part of the solution to the principal-agent problem. The failure to clearly understand the underlying preferences that determine the patients’ choices during clinical consultation may result in misperception of the patients’ utility function, which will, in turn, compromise the course of treatment and will not allow the design of contract to be optimized [13]. Thus, literacy as an input factor to bettering health outcomes is very important for both principals and agents in this regard.

Efficiency and equity theories were also reviewed in this study. These are the two main criteria which are usually employed in economic analysis to guide the way in which resources are being allocated and used. The desire for efficiency is derived from the desire to change and improve the world [14]. This implies that efficient and effective use of health expenditure increases the chances of a productive nation through health improvement. The analysis of health production and healthcare uses a concept of technical efficiency, which is based on the relationship between resource inputs and outputs [15]. Production can only be technically efficient when the greatest possible output is produced from the fewest inputs to manufacture a certain amount of output. An example of this can be made from a number of patients that can be treated in an out-patient clinic, which would depend on the available number of nursing staff and medicine available [14]. Economic efficiency, on the other hand, is concerned with the cost of inputs used to produce a certain amount of output [16]. Cost-effectiveness is one of the alternative labels of economic efficiency which is being achieved for a given amount of possible output with the lowest possible cost. Equity is an important policy objective in every healthcare system worldwide, as it is the most important criterion for the allocation of resources [17].

In the context of healthcare, equity means a fair distribution of health and healthcare amongst the population, and in addition, fairness should be included in the burden of financing healthcare [1]. Though equity and equality represent different terms with different meanings, equity is always defined with respect to equality and inequality because fairness and equality go together, though it may not always be fair to be equal [15]. Imagine a situation where both healthy and unhealthy people are receiving the same medical attention or an equal amount of healthcare. It would not be fair to make these two groups of individuals equal. However, as mentioned before, equity is always defined with respect to equality. It may be considered equitable to have people with equal needs to healthcare have the same access to it equally [16].

### Related Literature

There is limited number of studies that specifically focus on the impact that public health expenditure has on health outcomes in both developed and developing countries. However, with the available literature, there is, to some extent, consensus in terms of the need for improvement of public healthcare sector performance. Most developed countries like Italy have experienced problems with governance, disorganisation, and uncontrolled spending, and these have resulted in the failure of the systems they have put in place. The failure of the local government to carry out decisions which have been taken at national level caused disparities, which have in turn been reflected in poor health outcomes in Italy. Amongst competitive, rich countries, the USA has been reported to have the poorest health outcomes even though their health expenditure is twice as much as other developed countries. Another reported causality for poor US health outcomes is the scarcity of equipped and trained physicians who have the ability to utilise their advanced technological health equipment. It is important to focus on social determinants of health or factors that shape people’s health past their lifestyle choices and medical treatment [18]. This includes income, education, job security, working conditions, early child development, and many other social determinants of health.

Most of the challenges exhibited in developing countries, as revealed in literature, include a lack of finances to improve health, a lack of medical personnel (most medical personnel in Zimbabwe leave the country for better salaries) and also their health is bedeviled by the poor environment in which they live. Most countries in the developing world, such as Brazil, have been faced with outdated healthcare systems which affect their ability to carry out all health-related expectations of their citizens. Moreover, major issues in most of these countries include management issues and underfunding of health institutions. In addition to this factor, there is insufficient investment in health by countries in the developing world and this makes it impossible to carry out some of their legislative intents towards making healthcare a universal right [19].

South Africa is faced with an unequal distribution of healthcare services as well as challenges of disease burden and a limited number of medical personnel [3]. With respect to healthcare and healthcare outcomes, the literature suggests a need for improvement, especially in the public healthcare sector. The SAHR 2017 indicated an improved response to the HIV/AIDS prevalence in SA. However, there are still challenges that prevent improvement of the SA healthcare system, including governance, leadership, and accountability. Moreover, there is a need to improve the district and facility level as well as the overall planning and implementation of the health workforce. One other key challenge is the successful implementation of health policies, which when implemented have the potential to improve the South African health status. The ratio of physicians has also been identified as needing improvement in the South African healthcare system.

## 4. Methodology and Data

To examine the impact that public health expenditure has on health outcomes, this study adopted and modified Anyanwu’s model [8]. Anyanwu’s study investigated whether health outcomes have been improved by health expenditures in Africa. The underlying objective of that study was to determine whether or not the explanatory variables had a significant impact on health outcomes. The basic equation in the study examined the direct impact of health spending on health outcomes. It reflected the level of mortality and effectiveness of preventive care and also paid specific attention to maternal and child health. The equation is presented as follows:

(1) $$Hea_{it}=\alpha_{1i}+\beta_{1}\;1n(Heaexp_{it})\;+\beta_{2}\;1n(Ethnicfrac_{it})\;+\beta_{3}\;1n(Female_{it})\;+\beta_{4}\;\;1n(Urbanpop_{it})\;+\beta_{5}\;1n(y_{it})\;+\beta_{6}\;P1n(Physicians_{it})\;+\mu_{it}$$

where $Hea_{it}$ represents health outcomes such as the under-five mortality rate or infant mortality rate (outputs) while inputs are assumed to be $Heaexp_{it}$ (per capita health expenditure, i.e., variable of interest), $Ethnicfrac_{it}$ (index of ethnolinguistic fractionalization), $Female_{it}$ (female literacy rate), $Urbanpop_{it}$ (urban population as a measure of urbanization), $y_{it}$ (GDP per capita in international dollars), $Physicians_{it}$ (number of physicians per 100,000 population) and μ*_it_* is the error term.

The explained variable in this study is health outcomes (*Hea*) and it is explained by the shift in variables which are public health expenditure (*Phe)*, provincial gross domestic product (*GDP)*, human immunodeficiency virus (*HIV*), and the ratio of physicians per province (*RoP*). The model is specified as follows:

(2) $$Hea_{it}\;=\;\beta_{0}\;+\;\beta_{1}Phe_{it}\;+\;\beta_{2}GDP_{it}\;+\;\beta_{3}HIV_{it}\;+\;\beta_{4}RoP_{it}\;+\;\varepsilon_{it}$$

where $\beta_{1}$, $\beta_{2}$, $\beta_{3}$, and $\beta_{4}$ are coefficients of the variables. $\beta_{0}$ is a constant and $\varepsilon_{it}$ is the error term consisting of an idiosyncratic component and an unobserved heterogeneity component. The cross-sectional dimension *i* is to capture the nine South African provinces (Eastern Cape, Free State, Gauteng, KwaZulu-Natal, Mpumalanga, Limpopo, North West, Northern Cape and Western Cape) for the period (*t*) 2002 to 2016.

## 5. Estimation Results and Findings

### 5.1. Under-Five Mortality Rate Results

To answer the broader objective of this study as outlined in the introductory section, this study employed panel data analysis with three basic regression methods. Of these three (fixed effects (FE), random effects (RE) and pooled OLS), one was the appropriate estimator for the study and others were not, depending on the type of data or N and T dimensions employed in this study, all of which is presented in this section. In addition to the pooled OLS, FE, and RE, robust standard error estimates are presented towards the end of this section.

Moreover, the diagnostic check assisted in the identification of the correct, robust standard error estimator for this study. The regression results for pooled, FE, and RE estimators are presented in Table 1 below. The Breusch Pagan/Lagrange multiplier (BP/LM) was implemented to test the appropriate estimator between the RE and the pooled OLS. The BP/LM test for the random effects test displayed a test statistic of 13.11 and a *p*-value of 0.0001. These results led to the decision of rejecting the null hypothesis that the pooled OLS is the appropriate estimator for the study. This suggests that the BP/LM test favored the random effects model as the appropriate estimator. Moreover, to detect the appropriate estimator between the fixed effects and the random effects models, the study employed the Hausman test. The random effect regression is only consistent and efficient when errors are not correlated with explanatory variables. Thus, to detect whether errors are correlated with the regressor or not, the paper used the Hausman test. The Hausman test regression results showed a *p*-value of 0.0054, which is less than 5% (0.05) and a test statistic of 14.70. With the *p*-value being less than 5% (0.05), the null hypothesis that random effect is the appropriate model for the study could be rejected. Therefore, the fixed effects method became the appropriate estimator for the study. The time-invariant characteristic of the fixed effects model is one of the most important assumptions of the model, thus making it the best model for the study in this regard [20].

Although the fixed effect is the appropriate estimator, diagnostic checks were done to check if heteroscedasticity, serial, and cross-sectional correlation exist within the model. Therefore, the paper employed robust estimators (LSDV, SCC, and FGLS) to control for violation of the assumptions of the linear regression. 

Taking into account that the fixed effects is the appropriate estimator for the model, the public health expenditure variable (variable of interest) has the expected sign, and the *p*-value showed statistical significance. A 1 unit increase in Phe would lead to a 0.3% decrease in U5MR. In the FE model, public health expenditure is statistically significant at the 1% level of significance. As aforementioned, in the chosen FE model, the public health expenditure variable has an elasticity of −0.284 suggesting that a 1% increase in public health would lead to about a 0.3% decrease in the under-five mortality rate in South Africa, ceteris paribus. In other words, public health positively influences improvements in the under-five mortality rate. This finding is consistent with [6,8]. The elasticity found in this study is slightly lower than that which was found in [6], which was −0.350 and slightly higher than the elasticity which was found in [8], which was −0.21 in respect of African countries.

Although the ratio of physician’s variable has the expected sign, it was discovered to have a statistically insignificant impact on U5MR. This outcome shows that it seems like there is no statistically significant relationship between the ratio of physicians and the under-five mortality rate. This finding suggests that there are other factors that impact on the under-five mortality rate that play a greater role in the improvement of this health outcome than the ratio of physicians. An example of this would be the impact that public health expenditure has on the under-five mortality rate. Moreover, HIV and AIDS: Antenatal has the expected sign as depicted in Table 1 above. However, it was found to be a statistically insignificant influencer on the under-five mortality rate. In as much as HIV is an important factor, the impact could have been mitigated by different programs the government has adopted. Bor et al. (2013) mentioned that the prevention of mother-to-child transmission (PMTCT) program has a positive impact on health outcomes [6]. Figure 1 also presents that HIV has the expected negative sign but is not a driver of the under-five mortality rate, due to the strides that have been made by the South African government regarding the prevention of mother-to-child transmission (PMTCT) program. PMTCT is offered to 98% of South African public health facilities which resulted in a steady 2.7% decrease in mother-to-child transmission (MTCT) in 2011 [21].

### 5.2. Life Expectancy at Birth Results

Table 2 presents results for the life expectancy regressions. The Hausman specification test showed a *p*-value of 0.0007 and a *t*-value of 19.30 for the life expectancy at birth model. Moreover, the Hausman specification test failed to reject the fixed effects model as a proffered estimator to the random effects model. Public health expenditure (Phe) being the main explanatory variable for this study displayed statistical significance. In column three of Table 2 it is indicated that a unit increase in Phe would lead to a 0.09% increase in life expectancy at birth, holding all other variables constant. The significant relationship between public health expenditure and life expectancy at birth is inconsistent with the one explained in [6]. However, this finding is consistent with [8].

Considering the fixed effects model as the appropriate estimator for this study, the ratio of physician’s variable has the expected sign. However, it is a statistically insignificant influencer of life expectancy at birth. Moreover, although statistically insignificant, the regression results for life expectancy at birth under the FE model showed that a 1% increase in HIV and AIDS for total population would lead to a 0.04% decrease in life expectancy at birth. Regarding provincial GDP, it was discovered that there is a 5% significant relationship between GDP and the variable of interest as shown in Table 2 below. However, the coefficient sign is not as expected. This finding is inconsistent with the findings in [6].

It is interesting to discover that the chosen FE results reveal that although there is a statistical significance at 5% significance level for GDP, the sign is not as expected. This finding might be due to the indirect relationship between variables. Nevertheless, the results warranted further investigation into the relationship between life expectancy at birth and provincial GDP.

In summary, the results have shown that the under-five mortality rate is strongly influenced by public health expenditure. It is interesting to have discovered that the regression results showed no statistical significance between the ratio of physicians and the under-five mortality rate as this relationship has been reported [22,23] to be a positive relationship. Life expectancy at birth is influenced by public health expenditure at the 1% significance level. Though statistically insignificant, the ratio of physicians had the expected sign, and this finding also warrants further investigation.

### 5.3. Seemingly Unrelated Regression Results

The seemingly unrelated regression (SUR), Driscoll Kraay (SCC), least squares dummy variables (LSDV) and feasible generalized least of squares (FGLS) were employed for consistent and efficient inferences. To check for the appropriate estimator for the study, the Hausman specification test was employed, and it indicated that the fixed effects estimator is the appropriate estimator for this paper. To analyze the provincial impact of public health expenditure on health outcomes, the SUR was utilized. 

The main objective of this study was to assess the impact of health expenditure on health outcomes at the provincial level in South Africa. Therefore, Table 3 and Table 4 presents the LSDV and SUR findings with the province number in the first column, province name in the second column and the coefficient of health expenditure in the third to sixth columns. The use of panel data in this study allowed for the investigation of the impact of health expenditure on health outcomes both at an aggregated and disaggregated level in South Africa. Moreover, the use of the SUR allows for provincial differences considering that the provinces have different ways and governance in achieving the desired outcomes. This suggests that the provinces are different in terms of approach, practices, and governance in respect to health provision. Thus, the health expenditure impact is expected to vary across these different provinces. Due to the fact that the FE model can lead to an improper aggregation of heterogeneous effects, the SUR model in this study becomes relevant.

#### 5.3.1. Under-Five Mortality Rate

In Table 3 above, LSDV and SUR results are presented. These results are from different estimation techniques for robustness where the coefficients are basically the same but with slight differences across variation techniques. This table presents the results for the impact of public health expenditure at the provincial level specifically, with no other regressors. The statistical analyses were implemented using the STATA (version 14.02, StataCorp LLC, College Station, TX, USA) software. The SUR models and Stata package arranged the provinces from one to nine as follows: Eastern Cape (1), Free State (2), Gauteng (3), KwaZulu-Natal (4), Limpopo (5), Mpumalanga (6), Northern Cape (7), North West (8) and Western Cape (9).

With the consideration that the constant is included in the model, LSDV dropped the output for province (1) in the regression process as depicted in Table 3 above. The results show that Eastern Cape (EC), Free State (FS) and Limpopo were neither having the expected sign nor significant responsive to public health expenditure to reduce the level of U5MR. According to the Eastern Cape Department of Health: Annual Report 2016/17, one of the challenges encountered by communities in the province is access to health facilities due to the rural nature and the terrain of the province which has unreachable areas [24]. The Department of Health further reported that road infrastructure in the EC is in a very poor condition, especially the eastern and northern areas, due to underdevelopment and imbalances of the past.

#### 5.3.2. Life Expectancy at Birth

It can also be noted in Table 4 below that the number of observations has been reduced due to the same situation mentioned in the previous section. Table 4 presents the SUR regression results for life expectancy at birth for provincial individual analysis. It is evident from the SUR regression results that public health expenditure negatively affects life expectancy in the Eastern Cape and Mpumalanga. Moreover, public health expenditure is insignificant to life expectancy in these provinces. The reason for these findings might be linked to the reasons discussed in the previous section about the health conditions in the EC and MP province.

On the other hand, Table 4 shows regression results for Western Cape, Northern Cape, North West, Limpopo, and KwaZulu-Natal that are both positive and significantly explain the impact that public health expenditure has on life expectancy at birth. The regression results for Gauteng show the expected sign but no statistical significance between public health expenditure and life expectancy at birth. For these provinces, it means that a unit increase in public health expenditure would lead to a 0.04%, 0.08%, 0.1%, 0.22%, and 0.38% increase in life expectancy respectively. To some extent, these findings are consistent with [6].

## 6. Conclusions 

Policy recommendations and implications are drawn from the empirical results of this study as presented in the preceding section. Empirical findings in this regard indicated that the impact of public health expenditure on health outcomes varies across provinces based on the responsiveness of investigated factors and governance. The South African healthcare sector adopted strategies that include limiting personnel numbers, prioritizing primary healthcare, and a temporary reduction on infrastructure spending [5]. Limiting personnel numbers has the potential of creating more problems regarding improving health outcomes as a shortage of staff will be experienced in most public healthcare facilities. It is important for policymakers and the government to find and implement a policy that balances the demand and supply for medical services in public sectors. Limiting personnel numbers leads to excess demand, which in turn will lead to long-time lags between patient arrival and service delivery, which might also lead to loss of life to some extent. It is therefore important to find a balanced ratio of physicians per population.

Just as in the US healthcare system, the South African healthcare system faces challenges that include inefficiencies, healthcare quality, access, and results [25]. These are serious issues that the policymakers and governments need to address in order to improve the South African healthcare system, particularly in terms of access to basic healthcare and results. The South African healthcare system needs reformation that will help deliver better care at lower costs without disparities from one health organization (private healthcare) to another (public healthcare).

Most reported under-five mortality rate cases are due to the prevalence of HIV and AIDS, pneumonia and diarrhea. The prevention of mother-to-child transmission has proved to be one of the most significant ways of decreasing U5MR in SA [25]. Policies in this regard should be centered around further strengthening the PMTCT program at the provincial level. The National Department of Health (2012) recommended that mothers who are HIV positive should breastfeed exclusively [26]. Moreover, the mother and the baby should receive antiretroviral treatment or prophylaxis. In this regard, research [26,27] has shown that when antiretroviral treatment or prophylaxis is used by either the mother or the baby, HIV transmission through breastfeeding is reduced.

The ability to reduce U5MR represents an important health sector and societal goal at a global and national level [28]. The policymakers and government require accurate and complete data on the number of factors that result in child deaths. This enables them to plan and monitor child health and child service delivery [26]. The latter recommends that South Africa adopts a vital registration system that records both births and deaths on a continuous basis. This system helps to clearly monitor under-five mortality rates and collects accurate data on births and deaths of children within this age group, pointing to the causes of death on a continuous basis. There is a high proportion of child deaths that occur outside healthcare facilities [26]. This is a serious problem for a nation whose goal is to provide primary healthcare for all citizens. In this regard, the policymakers and National Department of Health should work towards making sure that healthcare services are made available in every region of South Africa to avoid deaths and illnesses unaccounted for.

In most developing countries, a significant proportion of death that breeds reduced life expectancy is due to non-communicable diseases such as hypertension, cerebrovascular accident, coronary heart disease, diabetes mellitus, chronic renal disease, and cancer, among others [29]. The latter implies that the growing prevalence of NCDs is mainly due to western-style diets and sedentary living which is worsened by inadequate nutrition, education, high prevalence of low birth weight, poor health services, lack of tobacco control, among others. In order to combat the decreasing life expectancy that is caused by NCDs, the community, the government and the health planners need to put equal effort to address relevant NCDs. Life expectancy can be improved through nutrition education, regular physical exercise, improved environmental planning, and development [25].

The key factor that affects life expectancy, as alluded to by other scholars [29], is economic growth and its impact on household income and purchasing capacity, particularly food. Public investment in social services such as health and education are also important determinants of improved life expectancy coupled with improved access to clean water and sanitation.

The most important finding in this study is that empirical results showed a significant relationship between public health expenditure and health outcomes in both models. The SUR individual provincial analysis in this regard showed that this impact varies across provinces. This suggests that there is something that certain provinces are doing well, that leads to a significant relationship between these variables that other provinces are not achieving. The National Department of Health and policymakers need to identify the similarities and differences between the provinces of South Africa in order to be able to enact policies that will benefit each province positively and that are tailored to their specific needs. While trying to find policies that work individually for each province, it is also important to factor in the issue of governance in each province because it is through proper governance that policies can be implemented successfully.

## Figures and Tables

**Table 1:** Regression results for under-five mortality rate (U5MR).

Variables	Pooled	Random	Fixed
Public Health Expenditure	−0.198 **	−0.203 **	−0.284 **
	(0.059)	(0.062)	(0.081)
GDP	−0.020	−0.022	−0.046
	(0.044)	(0.045)	(0.046)
HIV&AIDS: Antenatal	0.5196 **	0.489 ***	−0.465
	(0.188)	(0.202)	(0.355)
Ratio of Physicians	−0.2731 *	−0.258 *	0.103
	(0.140)	(0.148)	(0.201)
Constant	4.965 **	5.066 **	7.755 **
	(0.659)	(0.695)	(1.038)
r-Squared (R^2^)	-	0.1993	0.2728
f-Statistic	-	-	6.34
Wald (CHI)	43.43	39.95	-
*p*-Value	0.0000	0.0000	0.0000
CORR (u_1_ -, xB)	-	0	−0.7515
RHO	-	0.28645221	0.79366808
Observation	124	124	124
Provinces	9	9	9
Time Periods	14	14	14

**Source:** Author’s compilation, Data from South African Health Review (SAHR) (2002–2016) reports and Stats SA. *p* < 0.01 **, *p* < 0.05 ***, *p* < 0.1 *.

**Table 2:** Regression results for life expectancy.

Variables	Pooled	Random	Fixed
Public Health Expenditure	0.070 **	0.054 **	0.089 **
	(0.131)	(0.011)	(0.016)
GDP	−0.029 **	−0.034 **	−0.024 ***
	(0.009)	(0.009)	(0.009)
HIV&AIDS	−0.071 ***	−0.105 **	−0.038
	(0.027)	(0.025)	(0.031)
Ratio Of Physicians	0.047	0.749 **	0.001
	(0.033)	(0.029)	(0.042)
Constant	3.442 **	3.579 **	3.336 **
	(0.109)	(0.105)	(0.116)
r-Squared (R^2^)	-	0.5339	0.5725
f-Statistic	-	-	8.64
Wald (CHI)	151.52	137.26	-
*p*-Value	0.0000	0.0000	0.0000
CORR (u_1_ -, xB)	-	0	−0.4065
RHO	-	0.22305483	0.70723366
Observation	124	124	124
Provinces	9	9	9
Time Periods	14	14	14

**Source:** Author’s compilation, Data from SAHR (2002–2016) reports and Stats SA. *p* < 0.01 **, *p* < 0.05 ***, *p* < 0.1 *.

**Table 3:** Under-five mortality results for seemingly unrelated regression (SUR).

Province/Equation Number	Province Name	LSDV	FE-SUR	SCC-SUR	FGLS-SUR
1	Eastern Cape	-	0.595	0.595	0.595
		-	(0.621)	(1.013)	(0.498)
2	Free State	−0.276 *	0.210	0.210	0.210
		(0.147)	(0.308)	(0.482)	(0.247)
3	Gauteng	−0.485 **	−0.833 ***	−0.833 *	−0.833 **
		(0.123)	(0.328)	(0.411)	(0.263)
4	KwaZulu-Natal	0.139	−0.731	−0.731	−0.731
		(0.151)	(0.649)	(0.589)	(0.520)
5	Limpopo	−0.691 **	0.463	0.463	0.463
		(0.154)	(0.517)	(0.408)	(0.415)
6	Mpumalanga	−0.325 ***	−0.163	−0.163	−0.163
		(0.154)	(0.326)	(0.269)	(0.261)
7	Northern Cape	−1.417 **	−0.141	−0.141	−0.141
		(0.299)	(0.166)	(0.087)	(0.133)
8	North West	−0.402 **	−0.895	−0.895 *	−0.894
		(0.117)	(0.759)	(0.439)	(0.579)
9	Western Cape	−1.211 **	−0.985 ***	−0.385 **	−0.385 **
		(0.258)	(0.154)	(0.058)	(0.123)

**Source:** Author’s compilation from Stata (13) regression results. *p* < 0.01 **, *p* < 0.05 ***, *p* < 0.1 *.

**Table 4:** Life expectancy at birth results for SUR.

Province/Equation Number	Province Name	LSDV	FE-SUR	SCC-SUR	FGLS-SUR
1	Eastern Cape	-	−0.149	−0.149	−0.149
		-	(0.140)	(0.207)	(0.112)
2	Free State	0.022	0.135 *	0.135 *	0.135 **
		(0.029)	(0.063)	(0.065)	(0.050)
3	Gauteng	0.041	0.069	0.069	0.069
		(0.026)	(0.077)	(0.069)	(0.062)
4	KwaZulu-Natal	−0.087 **	0.375 ***	0.375 ***	0.375 **
		(0.027)	(0.151)	(0.134)	(0.121)
5	Limpopo	0.064 **	0.217 **	0.217 **	0.217 **
		(0.024)	(0.055)	(0.053)	(0.044)
6	Mpumalanga	0.073 **	−0.033	−0.033	−0.033
		(0.028)	(0.103)	(0.104)	(0.083)
7	Northern Cape	0.211 **	0.084 **	0.084 **	0.084 **
		(0.057)	(0.019)	(0.016)	(0.015)
8	North West	0.072 **	0.100 *	0.100	0.100 **
		(0.024)	(0.051)	(0.063)	(0.039)
9	Western Cape	0.146 **	0.038 ***	0.038 ***	0.038 **
		(0.039)	(0.016)	(0.016)	(0.013)

**Source:** Author’s compilation from Stata (13) regression results. *p* < 0.01 **, *p* < 0.05 ***, *p* < 0.1 *.
